# On Some New Local Anesthetics

**Published:** 1906-09

**Authors:** H. Braun

**Affiliations:** Translated for Buffalo Medical Journal, from Deutsch. Med. Wochenschrift, 1905. No. 42


### TRANSLATION.

### On Some New Local Anesthetics.

By PROF. DR. H. BRAUN.

Translated for Buffalo Medical Journal, from Deutsch. Med. Wochenschrift, 1905. No. 42.

BY means of a few researches upon his own body or upon the healthy, a person adapted for self observation may within a few hours get his bearings very accurately upon the local action of a new remedy. These simple researches, the method of which I have depicted in my manual (*Die Local Anästhesie,* etc., Liepzig, 1905), are harmless in the case of remedies whose general pharmacological qualities have already been investigated, and allow us for the most part the possibility of being able to state in respect to a given remedy submitted to us whether and in what direction practical researches will pay us or not.

The qualities which a local anesthetic must possess,—apart of course from the faculty possessed by many substances for anesthetisation,—are as follows:

1. It must in relation to its local anesthetic potency be less toxic than cocain. We may therefore speak of a relative and absolute toxicity. Most of the substances recommended as substitutes for cocain are indeed less toxic, possessing a slight absolute toxicity, but corresponding to this there is a slighter anesthetic action, so that the relative toxicity is not less than that of cocain. The relation of their toxicity to that of cocain may readily be determined by animal experiment, and it appears that this relation can be straightway applied to man. The importance of this first condition is much overestimated. One should not forget that most of the forms of application of cocain when properly carried out are free from danger. It is only in spinal anesthesia that cocain is surpassed by other remedies, and here the ill collateral effects are at least for the most part not dependent upon its toxicity in the ordinary sense of that term.

2. The remedy should not exert the least irritant action, nor cause the slightest injury to the tissues. Like cocain, tropacocain and eucain it must be absorbed without causing after effects at the site of application, as hyperemia of a disturbing grade, inflammation, infiltration or necrosis. Only then are we justified in assuming that the healing of the wound is not unfavorably influenced, and that no other injury occurs. Substances which have a strong acid or alkaline reaction are therefore to be excluded in advance, because they invariably cause local damage to the local tissues. Upon this important second condition very many new remedies have been wrecked.

3. The remedy must be soluble in water, and its solutions must be permanent to a certain extent. It is very desirable also that the solution should be sterilisable by some simple method. Cocain as is well known falls short in this respect.

4. The remedy must permit of a combination with suprarenin, without exerting any deleterious action on the vaso-constrictor action of the latter. All substitutes for cocain hitherto introduced do not fill this requirement; they either entirely abolish the action of suprarenin or cause notable disturbances. In order therefore to be able to utilize fully the local action of small doses of suprarenin, cocain alone can be combined with the latter.

In definite forms of application other things come into question. For application to mucous surfaces an anesthetic should have the power of rapid penetration. The anesthetising power is dependent upon this factor in the first degree. Just here cocain possesses an immense advantage over other substances. Finally the qualities necessary for a spinal anesthetic can not be reduced to a formula. The principal and collateral action of remedies when injected into the spinal canal are not as yet sufficiently known.

Within a short time the chemical houses have introduced three new local anesthetics,—namely, stovain, alypin and novocain. The latter alone is considered here:

NOVOCAIN

The chemical properties of this substance, which was discovered by Einhorn and has been placed at my disposal by the Farbwerke are here described for the first time. They are as follows: novocain is the monochlorhydrate of p-amino-benzoyl-diaethyl-amino æthanol with the formula:

```

        N H2

         |

         C1

   H|C       H|C

   H|C       H|C

         4C

         |

        COO.C2 H4.N(C2H5)2 H.C1.

```

The salt crystallises from alcohol in needles which melt at 156° C. It dissolves in water in proportion 1:1; and in cold alcohol 1:30 (the description and pharmacology are given word for word with Biberfield's account.) The author's own tests of novocain gave the following results:

1. 0.1% isotonic solution of novocain. Formation of a cutaneous wheal on the forearm. Injection painless. The anesthetic action like that of tropacocain, was of very short duration, and in from 3 to 5 minutes cutaneous sensibility returned. No hyperemia. The wheal vanished without leaving a trace.

2. 0.5% and 1% solutions novocain. Formation of cutaneous wheals. Injection painless. Duration of wheal anesthesia 15 minutes. Wheals vanished leaving no trace. No hyperemia.

3. 5% and 10% solutions novocain. Formation of wheals. Injection of 5% solution painless; 10% solution produced very slight irritation. Duration of anesthesia 17 and 27 minutes respectively. Very slight hyperemia at site of injection. Wheals vanished. No infiltration or tenderness remained.

4. 1% novocain solutions. 1 c.cm. injected simultaneously into forearm in region of superficial radial nerve. Soon after the skin over the injected area showed diminished sensibility. No distinct evidence that the peripheral nerve terminals were anesthetized.

5. 0.5% novocain solution. Constriction of little finger with rubber tube. Injection of 1 c.cm. solution circularly into the subcutaneous tissue of the first phalanx. After 11 minutes entire finger completely insensible. Rubber tubing removed. In 5 minutes sensibility had returned. No swelling or sensitiveness remained in finger.

We have to do therefore with a local anesthetic with a strong, yet in comparison with some others, a transitory action, like that of tropacocain. In order to obtain results comparable with those from cocain, it would be necessary to use concentrated solutions and large doses in proportion to the slight toxicity of novocain. However, this necessity is readily and successfully overcome by the addition of suprarenin to the novocain solutions.

6. 0.1% isotonic novocain solution. To 100 c.cm. added 5 drops 1:1000 suprarenin solution. Formation of cutaneous wheals on the forearm. Injection painless. Very marked anemia. Duration of anesthesia more than an hour. No reaction of any kind.

7. 1% novocain solution, each c.cm. of which contained 2 drops suprarenin solution 1:1000. Formation of wheals on forearm. Anesthesia extended far beyond the limits of wheals.

Duration nearly four hours. Marked suprarenin anemia, upon subsidence of which some after pain. No other reaction.

8. ½ c.cm. of the same solution injected beneath skin of forearm. The skin over the site of injection as well as in the course of the sensible nerves was anesthetic for 2 hours. Marked suprarenin action. No reaction.

9. ½% novocain solution with addition of a drop of suprarenin solution 1:1000 to each c.cm. One c.cm. injected beneath the skin of the first phalanx of the fourth finger. In 10 minutes finger anesthetic and anemic. Sensibility began to return in 65 minutes. Another hour elapsed before complete return of sensibility. No after pain.

The apparent superiority of novocain-suprarenin solutions to cocain-suprarenin solutions, at least for tissue injections, has been abundantly confirmed by clinical experience. The author has used novocain in 150 operations in which the cases were adapted for tissue injections. Among these are represented: The anesthetisation of the entire external nose (30 c.cm. solution, No. 2), the extirpation of a cancer of the scalp the size of the palm of the hand, including repair of the defect by skin transplantation from the arm, uranoplasty, staphylorrhaphy, enterostomy, laparotomy for tuberculous peritonitis, operation for inguinal hernia (Bassini's method), castration, hydrocele operation with removal of the tunica, and the like.

The majority of the operations were upon the hands and fingers for injury and suppuration, incisions, exarticulations of fingers, etc. There were numerous extractions of teeth. The anesthetisation of the inferior alveolar nerve and lingual nerve succeeded with novocain as well as with cocain. With 5 c.cm. novocain solution No. 4, the suprarenin being reduced to one half, the entire alveolar process of the upper jaw could be rendered anesthetic at once, which result cannot be accomplished with cocain. When the latter is used for wholesale extraction, two sessions are necessary.

The author has never seen any toxic collateral effects from novocain, although he has repeatedly used as much as 0.25gm. the maximum dose for mankind is doubtless somewhat larger than this. Local irritation was never noted. Concerning anesthetisation of mucous membranes as well as spinal anesthesia with novocain the author's experience has been too small to draw conclusions. With a 10% solution each c.cm. of which contains 2 drops of suprarenin sol. 1:1000 the nasal mucosa was rapidly and deeply anesthetised. In a few cases of spinal anesthesia 0.05 gm. with 3 drops suprarenin sol. 1:1000, good and prolonged anesthesia of the anal and perineal regions was obtained; and with 0.1 gm. with 6 drops suprarenin sol. 1:1000 the anesthetic area reached Poupart's ligament. In 2 cases there was slight meningeal irritation afterwards thought due to the supranenin, which was perhaps not indicated.

